# Time to recovery from severe acute malnutrition and its predictors among under five children admitted to therapeutic feeding units of general and referral hospitals in Tigray, Ethiopia, 2020: a prospective cohort study

**DOI:** 10.1186/s12887-023-04144-5

**Published:** 2023-06-26

**Authors:** Gebretsadkan Fisseha Kidane, Kidane Zereabruk, Woldu Aberhe, Abrha Hailay, Guesh Mebrahtom, Gebreamlak Gebremedhn Gebremeskel, Teklehaimanot Gereziher Haile, Desalegn Massa Teklemichael

**Affiliations:** 1 Department of Epidemiology, Tigray Regional Health Bureau, Mekelle, Tigray, Ethiopia; 2 Department of Adult Health Nursing, School of Nursing, College of Health Sciences and Comprehensive Specialized Hospital, Aksum University, Aksum, Tigray, Ethiopia; 3 Department of Maternity and Neonatal Nursing, School of Nursing, College of Health Sciences and Comprehensive Specialized Hospital, Aksum University, Aksum, Tigray, Ethiopia; 4 School of public health, College of Health Sciences and Comprehensive Specialized Hospital, Mekelle University, Mekelle, Tigray, Ethiopia

**Keywords:** Ethiopia, Severe Acute Malnutrition, Therapeutic feeding units, Time to recovery, Under five children

## Abstract

**Background:**

Across the globe, an estimated 16 million children under the age of 5 are affected by severe acute malnutrition. Children with severe acute malnutrition are nine times more likely to die than well-nourished children. In Ethiopia, 7% of children under five are wasted, and 1% of these are severely wasted. A prolonged hospital stay increases the incidence of hospital-acquired infections. The aim of this study was to assess the time to recovery and its predictors among children 6–59 months old with severe acute malnutrition admitted to therapeutic feeding units of selected general and referral hospitals in Tigray, Ethiopia.

**Methods:**

A prospective cohort study design was conducted among children aged 6–59 months admitted with severe acute malnutrition in selected hospitals in Tigray that have therapeutic feeding units. The data were cleaned, coded, entered into Epi-data Manager, and exported to STATA 14 for analysis.

**Result:**

Among 232 children followed in the study, 176 have recovered from severe acute malnutrition with a recovery rate of 54 per 1000 person-days observation and the median time to recovery was 16 days with an inter-quartile range of 8. In a multivariable Cox Regression, feeding plumpy nut [AHR 0.49 (95% CI 0.2717216-0.8893736)] and failing to gain 5 gr/kg/day for three successive days after feeding freely on F-100 [AHR 3.58 (95% CI 1.78837–7.160047)] were found to have an association with time to recovery.

**Conclusion:**

Despite the median time to recovery is shorter than what has been reported in a few studies, we can conclude that this could not let children avoid any possible hospital-acquired infections. The impact of staying in a hospital may also extend to the mother/caregiver in terms of the infection that they may acquire or the costs imposed on them.

## Background

Across the globe, an estimated 16 million children under the age of 5 are affected by severe acute malnutrition. This number is staggering, most importantly because children with severe acute malnutrition are nine times more likely to die than well-nourished children. Severe acute malnutrition is a major cause of death in children under five, and its prevention and treatment are critical to child survival and development [1].

Based on the 2019 Ethiopia Demographic Health Survey (EDHS), 7% of children under five are wasted, and 1% of these are severely wasted [2]. Mortality remains high among children with severe acute malnutrition, who often die because of several factors such as associated childhood co-morbidities, e.g., diarrhea, pneumonia, shock, and non-adherence to management protocols by health care professionals. Among the children, 66 (12.52%) were dead, and 357 (67.7%) were recovered at the end of follow-up [3].

Acute malnutrition is linked to higher risk of morbidity and mortality. It is also linked to poor growth and development, which contributes to stunting when episodes are persistent or frequent [4, 5]. A longitudinal study at Jima University Medical Center conducted to estimate the incidence rate of hospital-acquired infection revealed that a prolonged hospital stay (6.3 more days) was associated with hospital-acquired infection [6].

As confirmed by consecutive EDHS results, there is a decline in stunting, underweight, and wasting in children under five years of age. However, a stunting rate of 38% remains a great concern with the subsequent life-course impact of malnutrition on the long-term health of individuals and the socioeconomic development of the nation. There is a regional variation in malnutrition ranging from 15% in Addis Ababa to 46% in Amhara; seven of the regions have a rate greater than 30%, and the rate in Tigray was found to be 39%, which exceeded the national average. The national levels of wasting and underweight in children under five years of age were 10% and 24%, respectively, and in Tigray, 11% and 23%, respectively [7, 8].

The main causes of nutritional problems across all regions were food insecurity, low dietary diversity, low awareness on how to use the available resources at home, poor maternal and child feeding practices, poor sanitation, poor coordination of sectors, cultural practices and lack of resources [9].

Ending hunger, achieving food security and improved nutrition, and promoting sustainable agriculture are set to be some of the sustainable development goals. Conducting extensive studies on the magnitude and management of severe acute malnutrition by the health sector can provide insight into how to achieve this goal in collaboration with other sectors [10].

Predictors of time to recovery from severe acute malnutrition and treatment outcomes have been extensively studied retrospectively. However other equally important factors related to the mother, like, socio-demographic and practices/behaviors of child feeding have not been reported. Also, there are limited data on how long a child stays while on inpatient therapeutic feeding program. Additional, To the best of our knowledge, there is no prospective study done in Tigray region. Therefore, the aim of this study is to assess time to recovery and identify predictors of time to recovery among children with severe acute malnutrition followed in the therapeutic feeding units of general and referral hospitals, Tigray, Ethiopia.

## Methods

### Study setting and design

A prospective cohort study design was conducted among children aged 6–59 months admitted with SAM in selected general and referral hospitals in Tigray having therapeutic feeding units. Most catchment areas of each hospital are known to have districts that have been identified as hot spots for acute malnutrition by the Tigray regional health bureau. The cases were followed for a maximum of four weeks (28 days) and monitored on a daily basis starting from the date at which each patient is admitted to the therapeutic feeding unit until declaration of any outcome or if they are lost from follow-up.

### Inclusion and exclusion criteria

All malnourished children admitted to these hospitals within the study period were included in the study as they are admitted to the therapeutic feeding units. Children with severe acute malnutrition in the therapeutic feeding units within the study period who were transferred from other health facilities, or if they have any deformity that hinders in taking anthropometric measurements precisely were excluded from this study.

### Sampling

Sample size was computed using STATA, Version 14. In most of the literature reviewed weight was found to be significantly associated with recovery. The variability of 0.5 (SD) of covariate of interest was used, probability of recovery observed 0.824 and considering any particular outcome to be with 5% marginal error and 95% confidence interval of certainty (α = 0.05). Finally, the number of subjects needed to achieve a power of 80% and assuming no subject to withdraw from the follow up, the total sample size required was 232 with an estimated number of events (E) 191. From 16 general and referral hospitals, 8 of them were selected randomly (using simple random sampling) to be included in the study.

### Data collection techniques and tools

We developed a structured questionnaire in English, after reviewing relevant literature, looking at the treatment multi-chart, national SAM management protocol. Data were collected through an interview with mothers or caregivers of the selected children for characteristics related to them and their children through face to face at the health institution, carrying out physical examinations, collecting from laboratory results. Eight data collectors (clinical nurses) with one BSc. nurse supervisor in each hospital had a training for two days by the principal investigator regarding the objective of the study, how to measure each characteristic, how to complete the questionnaire and solve any problem that arose.

Trained ward attendants measured the nutritional status of the children. Height and weight were measured using the appropriate scale to the nearest acceptable precision (100 g for weight and 0.5 cm for height). On admission, all children were examined by the attending health professionals. In addition to carrying out appetite test, clinical evaluations were done to assess the complications and co-morbidities they had. Every aspect of the follow-up including extent of edema and medical complications were monitored by the trained ward attendants using the treatment multi-chart of each unit.

## Analytical strategies

The main outcome in this study was time to recovery from SAM. Individuals who are lost to follow-up, defaulted, died, non-responder or medical transfer at the end of the study period were censored. The out-come of each subject was dichotomized in to censored or recovered. Survival table analysis was used to estimate cumulative proportion of survival among children with SAM at different time intervals. Log Rank Test and Kaplan Meier Survival Curve were used to identify if there is a difference in survival experienced by different independent groups and estimate nutritional recovery time after initiation of treatment respectively.

Data were cleaned, coded and entered into Epi-data manager and exported to STATA 14 for analysis. Followed by carrying out exploratory data analysis to check the levels of missing values. Descriptive statistics was used to summarize child and care giver characteristics, determine recovery rate and estimate median time to recovery. Survival table was used to estimate cumulative proportion of survival among children with SAM at different time intervals. Kaplan Meier Survival Curve and log rank test were used to estimate nutritional recovery time and to compare survival functions of different groups respectively.

In order to identify independent predictors of time to nutritional recovery a bivariate and multivariate COX-Proportional hazard models were carried out. Adjusted hazard ratios with their 95% Confidence Interval (CI) were estimated and P-Value less than 0.05 were used to declare presence of significant association between time to nutritional recovery and covariates. hazard ratio, 95%CI and P-value were used to assess the strength of association and statistical significance.

To ensure data quality, all data collectors had a training to resolve potential problems that arose. The collected data should be edited and processed timely. The same standardized and timely calibrated measurement instruments were used to all study participants. Multiple sources of information like, questionnaire and direct measurements was used. 20% was considered to be the maximum limit for those lost to follow up cases. Making sure that scales are functioning correctly, children weighed consistently without clothes at about the same time each day and scales read to the nearest division of the scale (100 g, 0.5 cm for weight and height/length respectively).

## Result

### Mother/Caregiver and child characteristics

Of all mothers/care givers, 117(50.4%) were found to be at their 30–49 years of age. Among all mothers/care givers, 120(51.7%), 179(77.1%), 117(50.4%) of them were with no education, residing in rural areas and with house wife-based livelihood respectively (Table 1).

From the total participants, 126 (54.3%) were males. The majorities (45.3%), of these children were 12–23 months old. Among children enrolled in the study, 156(67.2%) and 201(86.6%) were on exclusive breast feeding while they were under 6 months old and fully vaccinated for their age respectively (Table 2).

### Admission Criteria, Medical Complications and Comorbidities identified

The most commonly detected medical complications during admission were diarrhea/dehydration (38.1%), hyperthermia (10.8%) and severe anemia (10.2%). Among all children admitted to the therapeutic feeding units, 111(63.1%) failed their appetite test and the remaining were admitted due to the medical complications they had and possible danger signs detected. Of all recovered children 99(56.3%) were non edematous. With regard to the comorbidities identified, 69(29.7%) and 15(6.5%) of the children admitted to therapeutic feeding units were found to have pneumonia and HIV/AIDS respectively.

### Time to recover from SAM and the recovery rate

A total of 176(75.9%) children recovered from severe acute malnutrition. The other outcomes declared are transferring to OTP (7.8%), death (7.8%) and defaulting from the program (6%). The proportion of children with no edema recovered from SAM was 50.9%. The median time to recovery was 16 days (IQR = 8) and the recovery rate was 54 per 1000 person-days observation. This rate indicated that, 54 children were recovering from SAM, as if 1000 malnourished children have been followed for a day.

**Fig. 1:**
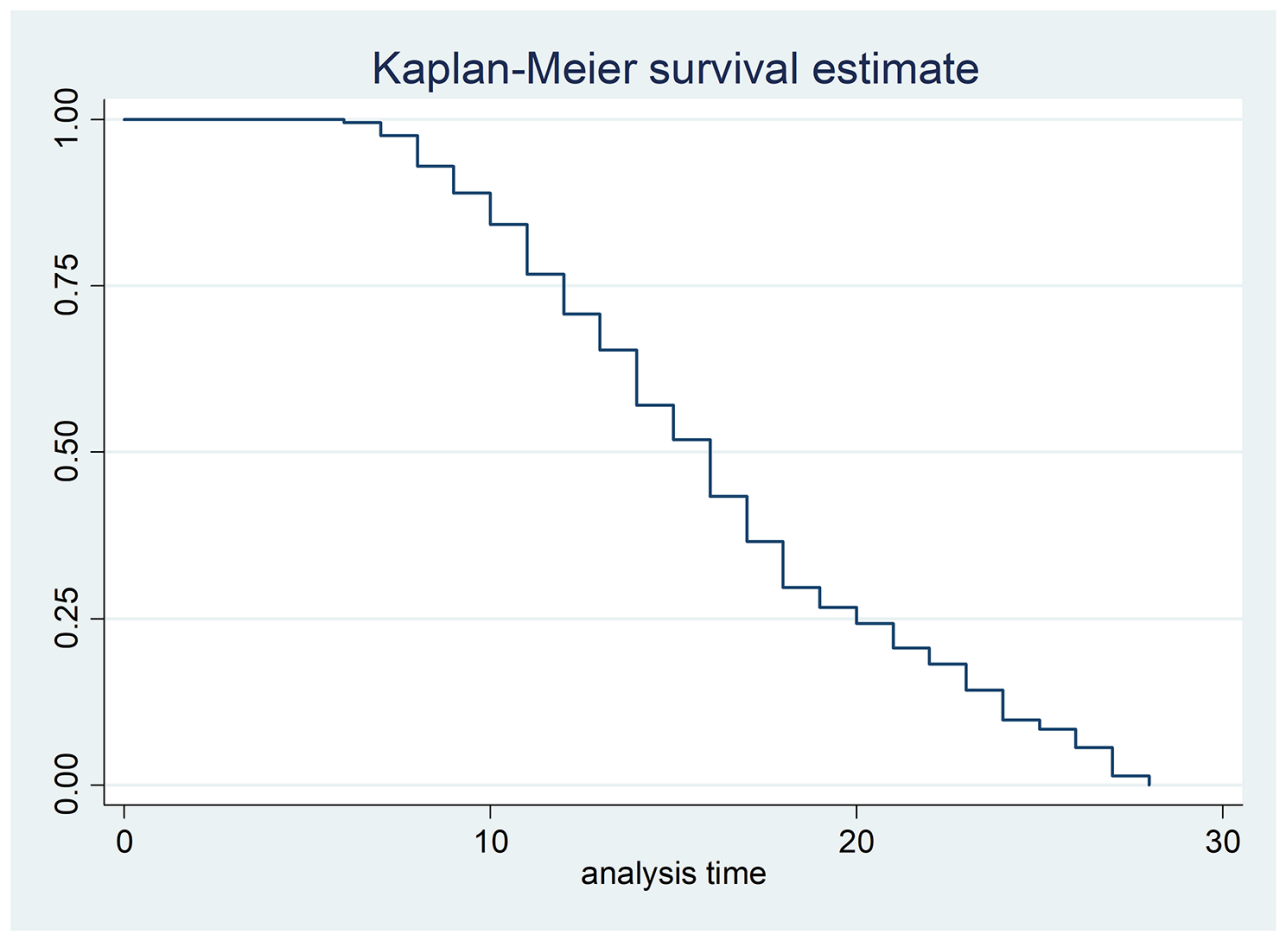
Overall Kaplan Meier curve of children with SAM, in therapeutic feeding units, hospitals, Tigray, Ethiopia

The overall Kaplan Meier curve illustrates that, there was a 75% probability of surviving by the end of the 12th day of their stay and the likelihood to recover from severe acute malnutrition by the end of the 20th day of their follow up was 25%. After the median time of recovery there was a gradual decline in the survival rate of children and no one stayed longer than 28 days (Fig. 1).

The survival table showed that, the cumulative probability of staying in the therapeutic feeding unit was 97.58% at 7th day, 57.03% at the 14th day, 20.64% at the 21st day and no one stayed at the end of the 28th day of his/her follow up (Table 3).

### Comparison of time to recovery among different groups

**Fig. 2:**
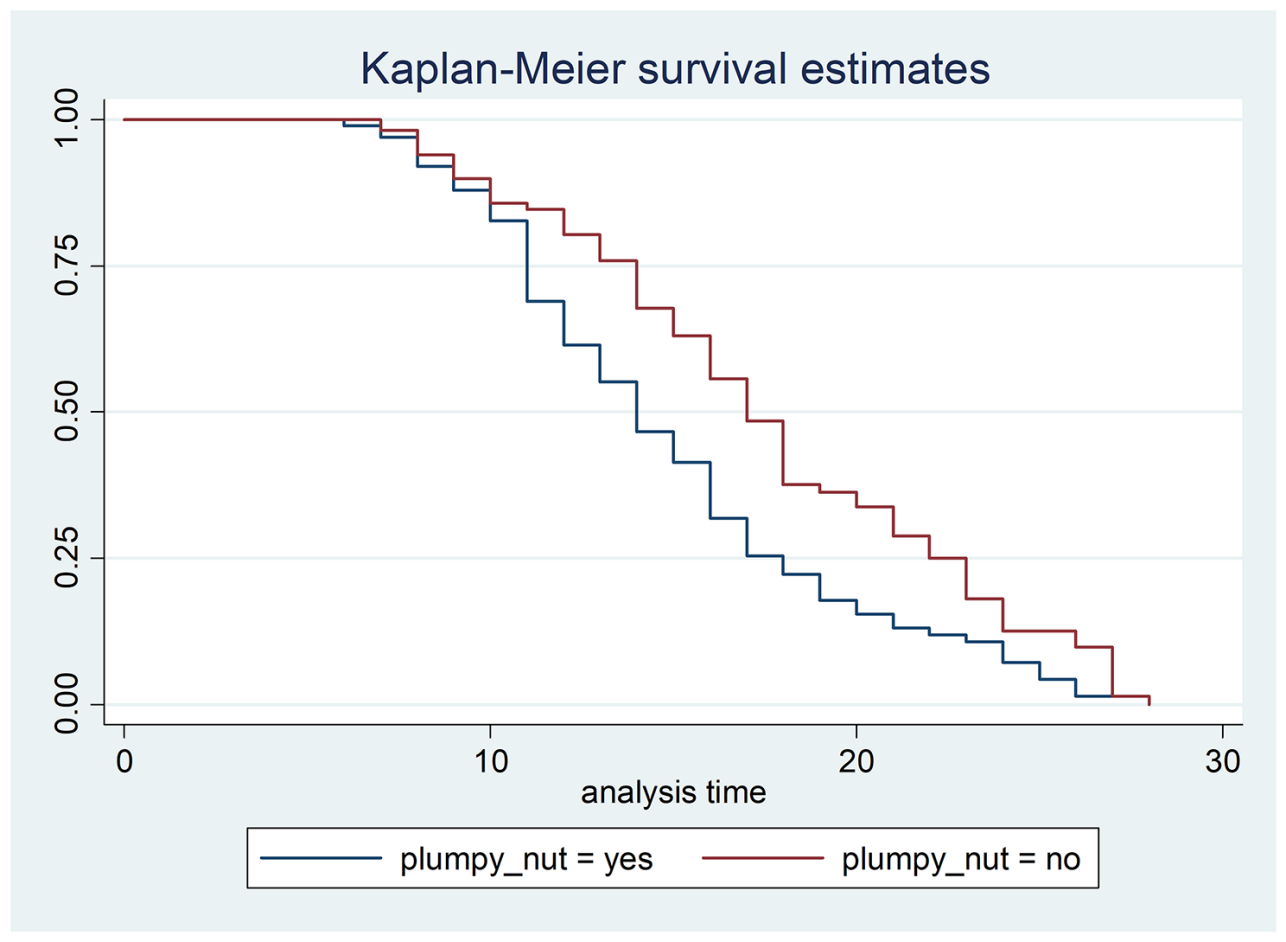
Kaplan Meier curve of children with SAM, in therapeutic feeding units of hospitals, Tigray, Ethiopia

There have been variations in the estimated median time to recovery and survival functions of children with respect to different grouping variables. Children who fed plumpy nut along with F-100 or alone during the transition phase and phase-II were found to recover earlier than those who did not feed plumpy nut. The difference in survival functions was significant at a p-value of 0.0016, as proven by log rank test (Fig. 2).

**Fig. 3:**
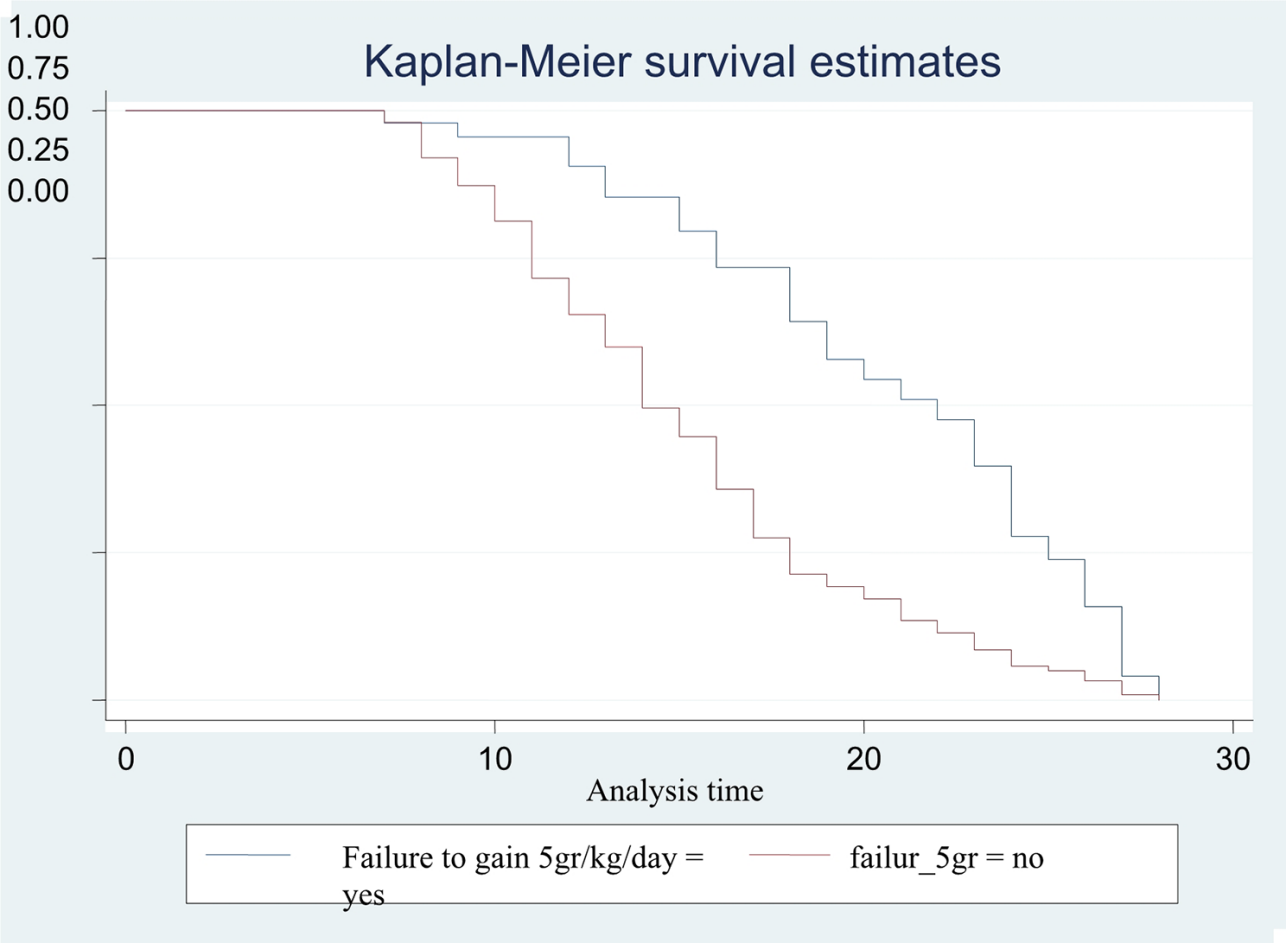
Kaplan Meier survival curve of children in TFU by weight gained during treatment, Tigray, Ethiopia

The Kaplan Meier curve of failure to gain more than 5 g/kg/d for three successive days after feeding freely on F-100, indicated that, children gaining 5gr/kg/day have recovered mostly earlier than (with median time to recovery of 15days) their counterparts whose median time to recovery was 23 days (Fig. 3).

The log rank test was used to test statistically of the differences in survival function identified in the Kaplan Meier curve as well among other possible grouping variables. There were significant differences in the survival functions among any family planning method users, children who got antibiotics intravenously, those who fed breast milk exclusively while they were less than 6 months old, failing to gain 5 gr/kg/day for three successive days after feeding freely on F-100 and feeding plumpy nut respectively with their counter parts (Table 4).

### Predictors of time to recovery from severe acute malnutrition in children admitted to therapeutic feeding units

Nine variables which passed through the bivariate analysis with a p-value < 0.25, were included while running the multivariate Cox Regression. Among all variables, feeding plumpy nut and failing to gain 5 gr/kg/day for three successive days after the initiation of F-100 feeding were found to be significantly associated with the time to recovery from severe acute malnutrition (Table 5).

After conducting the multivariate Cox Regression, the overall Schoenfeld global test of the full model was applied to test the assumptions of proportional hazard objectively and has met the proportional hazards assumption (at p = 0.2269).

**Fig. 4:**
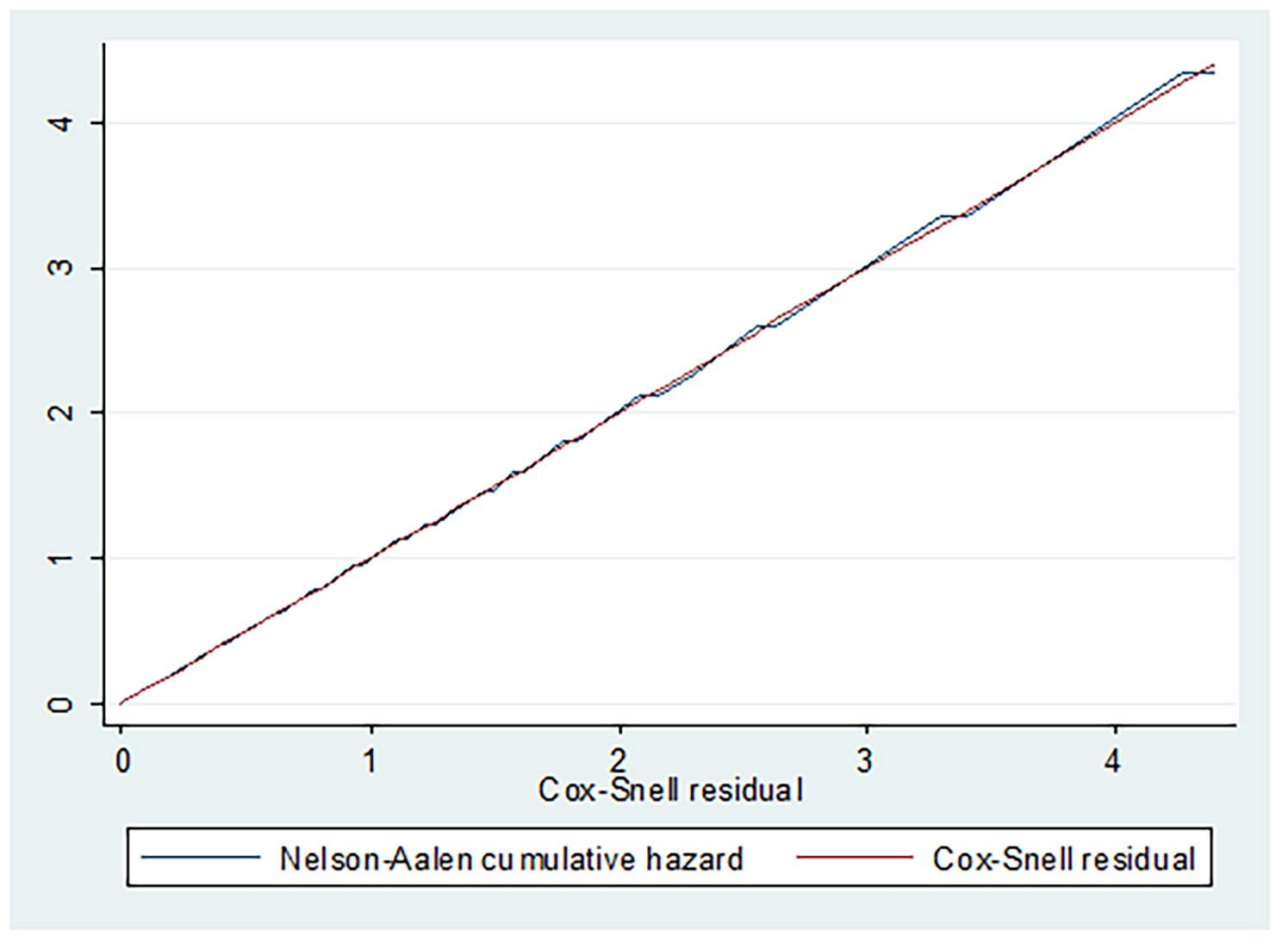
a cumulative hazard plot of Cox-Snell residuals for model fitness

Finally, to examine the overall fit of a cox model, we applied the Cox-Snell plot as if the hazard function followed the 45-degree line. It has revealed that the hazard function approximately has an exponential distribution with a hazard rate of one which indicated the model fitting the data well (Fig. 4).

After carrying out the multivariate Cox Regression, hazard ratios, 95%CI and P-value < 0.05 were used to assess the strength of association and statistical significance. The proportion of children who have recovered earlier than those who did not get plumpy nut was 50.8% [AHR0.49 (95%CI 0.2717216-0.8893736)]. The time to recover from severe acute malnutrition in children who failed to gain 5gr/kg/day for three successive days after feeding freely on F-100 was 3.58 folds longer time to recover from SAM than their counterparts [AHR 3.58 (95%CI 1.78837–7.160047)].

**Table 1:** Socio-demographic characteristics of mothers of children with SAM admitted to selected hospitals of Tigray, Ethiopia

Characteristics	Category	Proportion
Mother/care giver’s age group	15–19	6(2.6%)
	20–24	37(15.9%)
	25–29	67(28.88%)
	30–49	117(50.4%)
	> 49	5(2.1%)
mother/care giver’s educational status	no education	120(51.7%)
	primary	74(31.9%)
	Secondary	33(14.2%)
	above secondary	5(2.1%)
mother/care giver’s residence	Rural	179(77.1%)
	Urban	53(22.8%)
Ethnicity	Tigray	226(97.4%)
	Kunama	2(0.8%)
	Erob	
	Other	4(1.7%)
Marital status	Single	25(10.7%)
	married	193(83.2%)
	divorced	10(4.3%)
	widowed	4(1.7%)
Religion	Orthodox	197(84.9%)
	Muslim	17(7.3%)
	Protestant	
	Other	
mother/care giver’s occupation	housewife	117(50.4%)
	farmer	83(35.8%)
	Other	22(9.4%)
family size in number	<=5	124(53.4%)
	>=6	95(40.9%)
time elapsed when going to health institution in hours	<=2	182(78.4%)
	> 2	35(15.1%)
having time to prepare a food	Yes	169(72.8%)
	No	63(27.2%)
number of births given by mother/care giver	1–3	128(55.2%)
	4–6	95(40.9%)
	>=7	9(3.8%)
maternal/care giver’s age at first marriage (in years)	18	90(38.7%)
	>=18	142(61.2%)
history of using family planning methods	Yes	156(67.2%)
	No	76(32.8%)
main type of staple food used in the family	Maize	36(15.5%)
	Teff	99(42.67%)
	Millet	70(30.1%)
	Others	22(9.5%)

**Table 2:** Characteristics of children with SAM, admitted to therapeutic feeding units of selected hospitals, Tigray, Ethiopia

Variables	Category	Cured	Defaulter	Medical transfer	Transferred to OTP	Death	Total
Child’s age in months	6–11	41(23.3%)	2(14.3%)		1(5.6%)	4(22.2%)	48(20.7%)
	12–23	81(46%)	5(35.7%)	4(6.7%)	8(44.4%)	7(38.9%)	105(45.3%)
	24–36	46(26%)	6(42.9%)		9(50.0%)	7(38.9%)	68(29.3%)
	37–48	7(4%)	1(7.1%)	1(16.7%)			9(3.9%)
	49–59	1(0.6%)		1(16.7%)			2(0.9%)
sex of the child	Male	95(54.0%)	6(42.9%)	2(33.3%)	13(72.2%)	10(55.6%)	126(54.3%)
	Female	81(46.0%)	8(57.1%)	4(66.7%)	5(27.8%)	8(44.4%)	106(45.7%)
being orphan	Yes	6(3.4%)				1(5.6%)	7(3.01%)
	No	170(96.6%)	14(100.0%)	6(100.0%)	18(100.0%)	17(94.4%	225(97%)
Vaccination status	fully vaccinated	153(86.9%)	11(78.6%)	6(100.0%)	17(94.4%)	14(77.8%)	201(86.6%)
	not fully vaccinated	21(11.9%)	3(21.4%)		1(5.6%)	3(16.7%)	28(12.1%)
	not vaccinated at all	2(1.1%)				1(5.6%)	3(1.29%)
up to date vit A supplementation	Yes	142(80.7%)	12(85.7%)	5(83.3%)	15(83.3%)	15(83.3%)	189(81.5%)
	No	34(19.3%)	2(14.3%)	1(16.7%)	3(16.7%)	3(16.7%)	43(18.5%)
History of hospitalization	Yes	50(28.4%)	3(21.4%)	1(16.7%)	8(44.4%)	7(38.9%)	69(29.7%)
	No	126(71.6%)	11(78.6%)	5(83.3%)	10(55.6%)	11(61.1%)	163(70.3%)

**Table 3:** Survival table of children with SAM, in therapeutic feeding units of selected hospitals, Tigray, Ethiopia

Days	Beginning Total	Failed	Net Lost	Survivor Function	Standard Error	95%CI
1	232	0	3	1.0000		
2	229	0	4	1.0000		
3	225	0	6	1.0000		
4	219	0	5	1.0000		
5	214	0	3	1.0000		
6	211	1	5	0.9953	0.0047	0.9668–0.9993
7	205	4	8	0.9758	0.0107	0.9429–0.9899
8	193	9	2	0.9303	0.0180	0.8852–0.9582
9	182	8	4	0.8894	0.0222	0.8369–0.9258
10	170	9	4	0.8424	0.0260	0.7834–0.8864
11	157	14	0	0.7672	0.0305	0.7008–0.8208
12	143	11	1	0.7082	0.0329	0.6381–0.7673
13	131	10	4	0.6542	0.0346	0.5818–0.7171
14	117	15	1	0.5703	0.0363	0.4960–0.6378
15	101	9	1	0.5195	0.0368	0.4450–0.5887
16	91	15	0	0.4338	0.0368	0.3610–0.5044
17	76	12	0	0.3653	0.0359	0.2955–0.4353
18	64	12	2	0.2968	0.0342	0.2316–0.3647
19	50	5	1	0.2672	0.0332	0.2044–0.3338
20	44	4	0	0.2429	0.0324	0.1823–0.3083
21	40	6	0	0.2064	0.0307	0.1498–0.2695
22	34	4	2	0.1822	0.0294	0.1286–0.2432
23	28	6	0	0.1431	0.0271	0.0951–0.2006
24	22	7	1	0.0976	0.0233	0.0581–0.1491
25	14	2	0	0.0836	0.0220	0.0472–0.1331
26	12	4	0	0.0558	0.0185	0.0268–0.0999
27	8	6	0	0.0139	0.0097	0.0028–0.0449
28	2	2	0	0.0000		

**Table 4:** log rank tests of different groups of children with SAM, in selected hospitals, Tigray, Ethiopia

Variables		Cured	Log rank	p-value
Time elapsed while going to health institution	Yes	149	0.66	0.4157
	No	26		
	No	53		
Antibiotics given by intravenous	Yes	144	5.03	0.0249
	No	33		
Exclusively breast fed	Yes	117	6.35	0.0117
	No	58		
Feeding plumy nut	Yes	92	9.97	0.0016
	No	83		
Age at first marriage	< 18 Yrs	70	3.01	0.0827
	>=18yrs	105		
Comorbidity observed	Yes	66	0.42	0.5162
	No	109		
Failure to gain 5gr/kg/day while on F-100	Yes	52	43.78	0.0000
	No	123		

**Table 5:** Predictors of time to recovery of children with SAM, admitted to selected hospitals, Tigray, Ethiopia

Variables		*CHR	*AHR	p-value	95%CI
Marital status	Single	1.585858	0.9679679	0.899	0.5862716 − 1.59817
	Married	1	1		
	Divorced	1	1		
	Widowed	1	1		
	No	1	1		
Sites of waste disposal	Using a pit	0.7812503	0.776193	0.303	0.4795152 − 1.256427
	open field	1	1		
	Others	1	1		
IV antibiotics	Yes	1.53246	1.292317	0.366	0.7414192 − 2.252548
	No	1			
Fed Plumy nut	Yes	0.6381189	0.4915913	0.019	0.2717216 0.8893736
	No	1			
Failure to start losing edema at day-4	Yes	2.814735	0.8879964	0.826	0.3078144 2.56173
	No	1	1		
Fed breast milk exclusively	Yes	0.6847005	0.5702532	0.085	0.3007631 1.081212
	No	1	1		
Failure to enter phase-II on day-10	Yes	2.71032	1.212006	0.706	0.4455331 3.297081
	No	1	1		
Failure to gain 5gr/kg/day after F-100 feeding	Yes	2.893746	3.578381	0.000	1.78837 7.160047
	No	1	1		
Time at which complementary feeding started	< 6 months	1.120207	0.8390333	0.061	0.6985222 1.007809
	>=6months	1	1		

*CHR: Crude Hazard Ratio, *AHR: Adjusted Hazard Ratio

## Discussion

While prospective cohort study can provide valuable insights into the development of diseases and the effects of interventions, they also following participants can be challenging, particularly if they drop out of the study or are lost to follow-up. This can lead to missing data and reduced statistical power. These limitation and potential flaws, we had considered when interpreting the result. By using rigorous methods and addressing potential sources of bias, we ensure that our finding was robust and reliable.

We were able to have data that described possible characteristics of mothers/care givers through an interview conducted in the ward. As children on follow up were being monitored on a daily basis this study might have minimized any possible delays in ascertaining the outcome of interest.

The study provides information about the median time to recover from severe acute malnutrition and identify its predictors in Tigray, Ethiopia. Of the 232 children followed in this study, 176(75.9%) recovered, 14(6%) defaulted, 18(7.8%) died and 18(7.8%) were transferred to outpatient therapeutic feeding program. The maximum recovery time for the entire cohort was 28 days and no one stayed beyond that. The median time to recovery for the entire cohort was 16 days with an IQR of 8. The proportion of children whose outcome not known was 16.4%. Variables having a significant association, which are related to the care given and monitoring of the progress that the child had were feeding plumpy nut and failure to gain 5 gr/kg/day while feeding F-100.

The proportions in treatment outcomes of the entire cohort were within the acceptable range as per the international reference standards for SAM outcome indicators of children in therapeutic feeding units, which states, > 75% recovery, < 10% death, < 15% defaulters with 28 days mean length of stay. The median time to recovery in this study was consistent with the median recovery time reported in studies conducted in Jimma university, Hawassa University, Woldia hospital and in selected government health institutions in Amhara region [11–14].

However, there have been studies conducted in Bahirdar, felege hiwot hospital, Southern Ethiopia and Sekota hospital with longer median time of recovery than the current study [15–17]. The variation in median time to recovery among these study settings might be due to the difference in severity of cases they admitted, the quality services they deliver or availability of supplies used for the all types of cares.

With regard to the predictors of time to recovery, the proportion of children who have recovered earlier than those who did not get plumpy nut was 50.8% [AHR 0.49 (95% CI 0.2717216-0.8893736)]. This is in line with the study carried out in Bahirdar city, which revealed provision of plumpy nut as a predictor of time to recovery from SAM [18]. This might be due to the reason that, children who received plumpy nut have the possibility to achieve rapid weight gain and reach the discharge criteria earlier than their counterparts [19].

The time to recover from severe acute malnutrition in children who failed to gain 5 gr/kg/day for three successive days after feeding freely on F-100 was 3.58 times longer than their counterparts [AHR 3.58 (95% CI 1.78837–7.160047)]. This was consistent with the study conducted in Yekatit 12 hospital and might be due to the quality of care given and the way they are monitored per unit time of their stay [20].

The results of the published articles used retrospective cohort studies to identify the connections between risk factors and outcome, but their quality and completeness of data at a single point in time is limiting. Other studies used hospital-based cross sectional does not identify the causes or long-term effects. Prospective cohort study, providing robust evidence for causality, but they can be costly and time consuming to conduct. Prospective cohort study is generally considered the gold standard for studying the causes and long-term effects of disease.

Data on this study are needed to support under five children and mother or caregiver health programmers and policymakers and to formulate recommendations for future clinical practice and guidelines. Furthermore, studies have to be conducted to explore factors that might not have been identified in this study.

### Limitations of the study

As the design was a prospective cohort, it was difficult to know the outcomes of all children admitted mostly due to defaults and early transfer to OTP sites.

## Conclusion

This study has estimated the median time to recovery and also identified possible factors associated with times to recovery of children who have been on follow up during the study.

Despite that the median time to recovery is shorter than what has been reported in a few studies, we can conclude that this could not let children avoid any possible hospital acquired infections. The impact of staying in a hospital may also extend to the mother/care giver in terms of the infection that they may acquire or the costs imposed on them. Other members of the family including children might be left without any person who is responsible to provide a care. With regard to the predictors of time to recovery, had all the children been provided with the same care like giving plumpy nut along with the breast milk they fed or other therapeutic food and also been monitored their weight during follow up, they would have experienced the same survival probabilities during their stay.

Children who failed to gain the recommended weight during F-100 feeding were more likely to stay longer than their counterparts. We can also conclude that, the median time to recovery estimated in this study is longer than what has been reported in a few studies.

## Data Availability

This research is original, has not been submitted to or accepted for publication in any journal, and all information sources used in this study have been identified and thanked. The corresponding author will make all raw data produced or analyzed during the current investigation available upon request.

## Abbreviations

AHR: Adjusted Hazard Ratio
CHR: Crude Hazard Ratio
CI: Confidence Interval
EDHS: Ethiopia Demographic Health Survey
F-75: Formula 75
F-100: Formula 100
HSTP: Health Sector Transformation Plan
IU: International Units
IQR: Inter-Quartile Range
OTP: Out-Patient Therapeutic Program
RUTF: Ready to Use Therapeutic Food
SAM: Severe Acute Malnutrition
TFU: Therapeutic Feeding Unit

## References

[1] unicef., *severe acute malnutrition* 2015.

[2] Institute EPH (2019). Ethiopia mini demographic and health survey 2019: key indicators.

[3] Wagnew F (2018). Predictors of mortality among under-five children with severe acute malnutrition, Northwest Ethiopia: an institution based retrospective cohort study. Archives of Public Health.

[4] de Pee S (2015). Prevention of acute malnutrition: distribution of special nutritious foods and cash, and addressing underlying causes—what to recommend when, where, for whom, and how. FoodNutr Bull.

[5] Uauy R (2012). Global efforts to address severe acute malnutrition. J Pediatr Gastroenterol Nutr.

[6] Ali S, et al. Healthcare associated infection and its risk factors among patients admitted to a tertiary hospital in Ethiopia: longitudinal study. Volume 7. Antimicrobial Resistance & Infection Control; 2018. p. 2. 1. 10.1186/s13756-017-0298-5 PMC5755436 29312659

[7] EDHS E. Demographic and health survey 2016: key indicators report. The DHS Program ICF; 2016.

[8] Health FD. R.o.E.M.o., Health sector transformation plan (2015/16–2019/20). 2015, Ministry of Health Addis Ababa.

[9] Kennedy E (2015). Multisector nutrition program governance and implementation in Ethiopia: opportunities and challenges. FoodNutr Bull.

[10] Hawkes C, Popkin BM (2015). Can the sustainable development goals reduce the burden of nutrition-related non-communicable diseases without truly addressing major food system reforms?. BMC Med.

[11] Jarso H, Workicho A, Alemseged F (2015). Survival status and predictors of mortality in severely malnourished children admitted to Jimma University Specialized Hospital from 2010 to 2012, Jimma, Ethiopia: a retrospective longitudinal study. BMC Pediatr.

[12] Fikrie A, Alemayehu A, Gebremedhin S (2019). Treatment outcomes and factors affecting time-to-recovery from severe acute malnutrition in 6–59 months old children admitted to a stabilization center in Southern Ethiopia: a retrospective cohort study. Ital J Pediatr.

[13] Chane T (2014). Treatment outcome and associated factors among under-five children with severe acute malnutrition admitted to therapeutic feeding unit in Woldia hospital, North Ethiopia. J Nutr Food Sci.

[14] Baraki AG (2020). Time to recovery from severe acute malnutrition and its predictors: a multicentre retrospective follow-up study in Amhara region, north-west Ethiopia. BMJ open.

[15] Desyibelew HD, Fekadu A, Woldie H (2017). Recovery rate and associated factors of children age 6 to 59 months admitted with severe acute malnutrition at inpatient unit of Bahir Dar Felege Hiwot Referral hospital therapeutic feeding unite, northwest Ethiopia. PLoS ONE.

[16] Gebremichael DY (2015). Predictors of nutritional recovery time and survival status among children with severe acute malnutrition who have been managed in therapeutic feeding centers, Southern Ethiopia: retrospective cohort study. BMC Public Health.

[17] Desta K (2015). Survival status and predictors of mortality among children aged 0–59 months with severe acute malnutrition admitted to stabilization center at Sekota Hospital Waghemra Zone. J Nutr Disord Ther.

[18] Asres DT, Prasad RP, Ayele TA (2018). Recovery time and associated factors of severe acute malnutrition among children in Bahir Dar city, Northwest Ethiopia: an institution based retrospective cohort study. BMC Nutr.

[19] Gebremichael DY (2015). Predictors of nutritional recovery time and survival status among children with severe acute malnutrition who have been managed in therapeutic feeding centers, Southern Ethiopia: retrospective cohort study. BMC Public Health.

[20] Adimasu M et al. Recovery time from severe acute malnutrition and associated factors among under-5 children in Yekatit 12 Hospital, Addis Ababa, Ethiopia: a retrospective cohort study. Epidemiol health, 2020. 42. 10.4178/epih.e2020003 PMC7056942 32023778

